# Development of a novel TLR8 agonist for cancer immunotherapy

**DOI:** 10.1186/s43556-020-00007-y

**Published:** 2020-09-10

**Authors:** Yuxun Wang, Heping Yang, Huanping Li, Shuda Zhao, Yikun Zeng, Panpan Zhang, Xiaoqin Lin, Xiaoxiang Sun, Longsheng Wang, Guangliang Fu, Yaqiao Gao, Pei Wang, Daxin Gao

**Affiliations:** Shanghai Denovo Pharmatech Co., Ltd., 576 Libing Road, Shanghai Zhangjiang High-Tech Park, Pudong New District, Shanghai, 201203 China

**Keywords:** TLR8, Innate immunity, Cancer, Immunotherapy

## Abstract

Toll-like receptors (TLRs) are a family of proteins that recognize pathogen associated molecular patterns (PAMPs). Their primary function is to activate innate immune responses while also involved in facilitating adaptive immune responses. Different TLRs exert distinct functions by activating varied immune cascades. Several TLRs are being pursued as cancer drug targets. We discovered a novel, highly potent and selective small molecule TLR8 agonist DN052. DN052 exhibited strong in vitro cellular activity with EC50 at 6.7 nM and was highly selective for TLR8 over other TLRs including TLR4, 7 and 9. DN052 displayed excellent in vitro ADMET and in vivo PK profiles. DN052 potently inhibited tumor growth as a single agent. Moreover, combination of DN052 with the immune checkpoint inhibitor, selected targeted therapeutics or chemotherapeutic drugs further enhanced efficacy of single agents. Mechanistically, treatment with DN052 resulted in strong induction of pro-inflammatory cytokines in ex vivo human PBMC assay and in vivo monkey study. GLP toxicity studies in rats and monkeys demonstrated favorable safety profile. This led to the advancement of DN052 into phase 1 clinical trials.

## Introduction

Human immune defense system comprises both innate and adaptive immune pathways [1]. Most of the targets drugged by the recently approved cancer immunotherapeutic agents including the immune checkpoint proteins PD-1, PD-L1 and CTLA-4 function in adaptive immune pathways [2, 3]. In contrast, targets involved in the innate immune pathway had been under-developed [4, 5]. Innate immunity acts as the body’s first line of immune defense. Drugs targeting innate immunity hold potential for more rapid and broader spectrum anti-cancer effect than adaptive immunity. Furthermore, combinations of drugs targeting innate and adaptive immunity are expected to produce strong synergistic efficacy owing to their complementary nature as body’s immune defense [6]. Toll-like receptors (TLRs) are a family of proteins that recognize pathogen associated molecular patterns (PAMPs). Their primary function is to activate innate immune responses while they are also involved in facilitating adaptive immune responses [7]. Different TLRs differ in their expression in various target cells and exert distinct functions by activating varied immune cascades [7–9]. In the TLR family, several TLRs have been studied as cancer drug targets such as TLR2, 4, 7, 8 and 9 [7, 10–14]. Some of them are being drugged by conventional small molecule modality while others are being targeted by unconventional molecules such as oligonucleotide agonists for TLR9 [12, 13]. We reasoned that among different TLRs amenable to small molecule modality, specific targeting TLR8 would be advantageous. First, from the efficacy perspective, TLR8 has been shown to be necessary and sufficient to reverse the immune suppressive function of Treg cells leading to strong tumor inhibition [15–19]. TLR8 activation has also been shown to induce apoptosis of MDSCs [20]. MDSCs are another major type of cells that suppress immune response. Apoptosis of MDSCs can lead to activated and enhanced immune response to tumors. Treg and MDSC are the major cause of failure in cancer immunotherapy [20–22]. Therefore, reversing Treg and MDSC mediated immune suppression by activating TLR8 can elicit potent immune response resulting in strong anti-cancer effect [15, 23]. Moreover, TLR8 has been shown to induce terminal differentiation-mediated tumor suppression in acute myeloid leukemia (AML) [24]. Second, from safety perspective, TLR8 agonists have better safety profile allowing systemic administration whereas targeting other TLRs such as TLR4, 7, TLR7/8 dual, TLR9 appear to be less tolerable when dosed systemically and are mainly dosed locally through intra-tumoral injection or limited to topical use [10, 12]. For example, imiquimod, also known as Aldara, an approved drug predominantly targeting TLR7 is used as a topical drug because it is too toxic to use systemically, which dramatically limits its clinical application [25–27]. Systemic administration of TLR7 agonists has been explored using relatively low doses or prodrug form to reduce their systemic toxicity. However, further optimization of their dosing regimen and the compounds is necessary before systemic dosing can be applied in the clinic [28–30]. TLR7/8 dual agonists are being developed and also administered by intra-tumoral injection [31, 32]. Motolimod is generally known as a TLR8 agonist [33]. However, consistent with literature reports, we have found motolimod has weak activity on TLR7 in addition to its agonistic activity over TLR8 indicating it is in fact a TLR7/8 dual agonist though it predominantly targets TLR8 [34]. Motolimod is administered subcutaneously, a systemic dosing route [35, 36]. However, the clinical doses used for motolimod were relatively low and its efficacy appeared to be limited suggesting motolimod had a narrow therapeutic index [35, 36]. Nevertheless, unlike other TLR7/8 dual agonists, motolimod can be dosed systemically suggesting its weaker activity on TLR7 relative to other TLR7/8 dual agonists might contribute to its improved tolerability. TLR9 agonists are oligonucleotide based molecules that are distinct from conventional small molecules and are also dosed by intra-tumoral injection [13]. Taken together, among different TLRs, specific targeting TLR8 is expected to be advantageous considering both efficacy and safety characteristics. However, there are currently no approved drugs for TLR8 selective agonists. As described above, motolimod, a known TLR8 agonist, is in fact a TLR7/8 dual agonist albeit its TLR7 activity is much weaker than its activity on TLR8 based on in vitro cell-based assays [34]. Motolimod is currently in phase 1/2 clinical development for cancer indications [33, 35–40]. Another known TLR8 agonist in clinical development is selgantolimod (GS-9688). Interestingly, GS-9688 also has activity over TLR7 in addition to its predominant activity on TLR8 (Daffis, et al. EASL 2017 Amsterdam Netherlands). GS-9688 is now in Ph 2 development for chronic hepatitis B (hepatitis B infection) indication. GS-9688 is administered orally. However, the bioavailability of GS-9688 is extremely poor restricting its exposure to the gut, which is purposely designed to avoid its severe toxicity caused by systemic exposure (Daffis, et al. EASL 2017 Amsterdam Netherlands).

Therefore, there is an urgent need for a more selective TLR8 agonist that would distinguish itself from other TLR agonists. Through structure-based drug design, we discovered a novel, highly potent and selective small molecule TLR8 agonist with little activity on TLR7, namely DN052 for cancer indications. The detailed chemical structure and synthesis of DN052 and related compounds were described in our published patent 10,669,252. Since motolimod is a closely related TLR8 agonist in clinical development for cancer indications we included motolimod as a reference benchmark molecule in our study. Here, we present strong evidence demonstrating DN052 is a novel TLR8 selective agonist differentiated from other known TLR8 agonists and DN052 possesses excellent drug-like properties and is advancing in phase 1 clinical development.

## Results

### DN052 was a novel, potent and selective TLR8 agonist

**Fig. 1:**
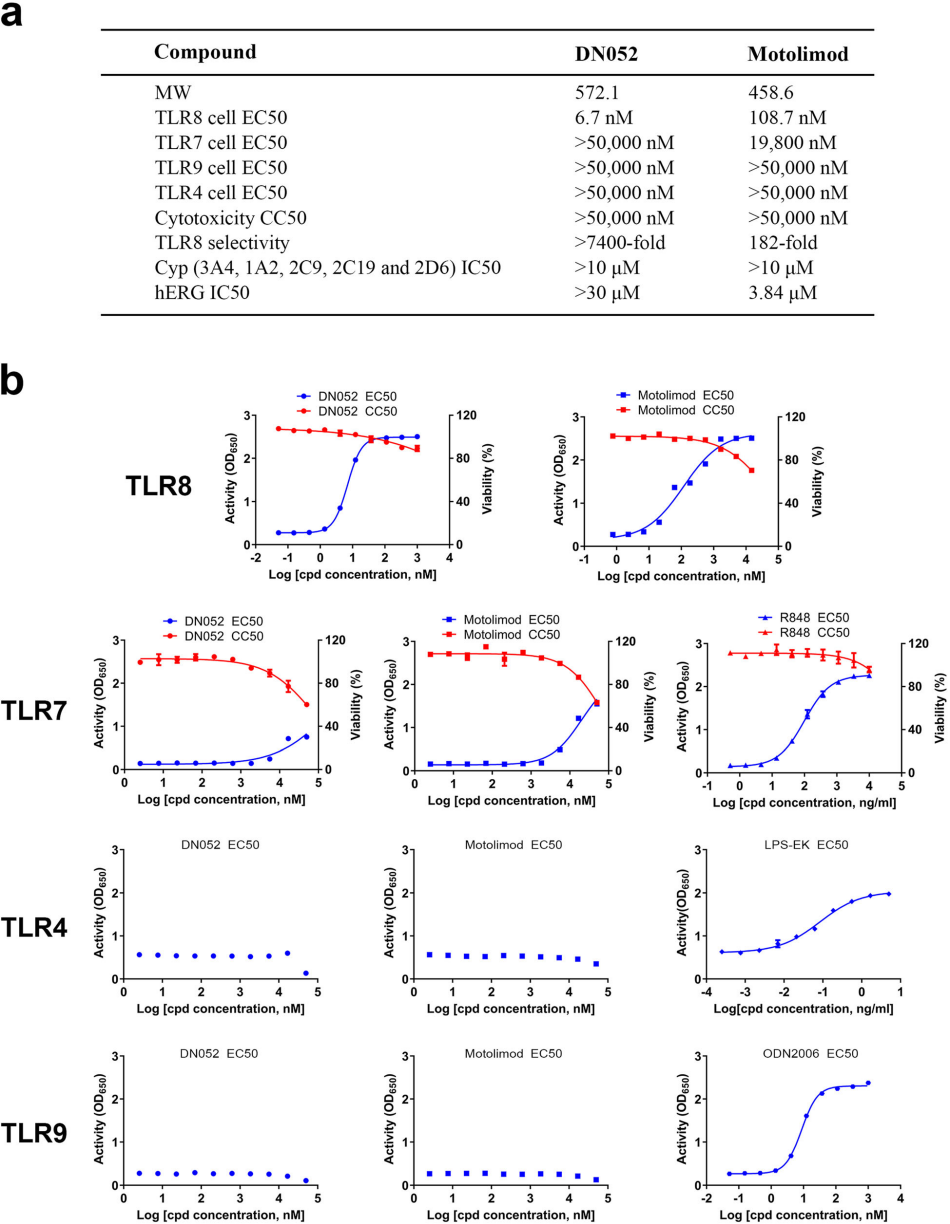
In vitro profiles of DN052. (**a**) Differentiated parameters of DN052. The molecular weight (MW) of DN052 and motolimod are shown. The detailed chemical structure and synthesis of DN052 were described in our published patent 10,669,252. (**b**) DN052 was potent and selective in cell-based assays. DN052 was evaluated for its agonistic effect on TLR8 and selectivity over TLR4, 7 and 9 in HEK-Blue™ TLR cells and the data was presented as EC50 (50% effective concentration). The potential effect of DN052 on cell proliferation was tested in HEK-Blue™ TLR7 and 8 cells in parallel and the data was presented as CC50 (50% cytotoxicity concentration). Motolimod was used as a reference compound. The agonists (LPS-EK, R848 and ODN2006) known to activate each of the TLRs were used as positive controls in TLR4, 7 and 9 assays, respectively. DN052 was highly selective for TLR8 over TLR4, 7 and 9. DN052 and motolimod had little effect on cell proliferation

The in vitro activity of DN052 and motolimod was determined in cell-based assays (Fig. 1). The EC_50_ of DN052 in hTLR8 agonist activity was 6.7 nM. The EC_50_ of the reference compound motolimod was 108.7 nM. The results indicated DN052 was about 16-fold more potent than motolimod in activating TLR8 in vitro. The average CC_50_ values of DN052 and motolimod were greater than 50 μM suggesting both compounds did not have substantial non-specific cell killing activity (Fig. 1a and b). To evaluate the selectivity of DN052 for TLR8 over several other related TLRs including TLR4, TLR7 and TLR9, cell-based assays in HEK-Blue™ hTLR4, hTLR7 or hTLR9 cell line expressing the corresponding human TLR and SEAP reporter gene was used. The result showed that the positive control compounds (LPS-EK, R848 and ODN2006) for TLR4, 7 and 9, respectively, were all active in the respective assays (Fig. 1b). In contrast, the EC_50_ of DN052 in the hTLR4, hTLR7 and hTLR9 agonist activity assays were greater than 50 μM, the highest concentration used in the assays, indicating DN052 was highly selective for TLR8 with little activity over TLR4, TLR7 or TLR9 (Fig. 1a and b). The EC_50_ of motolimod in the hTLR4 and hTLR9 agonist activity assays were greater than 50 μM. The EC_50_ of motolimod in the hTLR7 agonist activity assay was 19.8 μM indicating motolimod had weak activity over TLR7 in addition to its predominant agonistic activity for TLR8 (Fig. 1a and b).

To assess potential off-target effects of DN052 in comparison to motolimod, the Cerep screen was conducted for DN052 and motolimod in receptor binding, enzyme and uptake assays involving 44 targets. Results showing an inhibition or stimulation higher than 50% are considered to represent significant effects of the test compounds. As shown in Supplementary Fig. 1 and 2, overall, DN052 and motolimod shared similar profiles with DN052 displaying fewer off-targets than motolimod. Motolimod showed 59.2% against potassium channel hERG suggesting motolimod might have potential cardiac toxicity at high concentration. In contrast, DN052 did not show significant effect on the potassium hERG in the Cerep screen suggesting DN052 may be safer than motolimod when used at high concentrations. Of note, this Cerep screen was performed with 10 μM compounds which was a very high concentration unlikely to be used in pharmacology studies. In addition, motolimod has advanced to clinical phase 2 trials without report of unacceptable adverse events [35, 36]. Taken together, the off-target results supported further development of DN052.

### DN052 had favorable drug metabolism and pharmacokinetic profiles

**Fig. 2:**
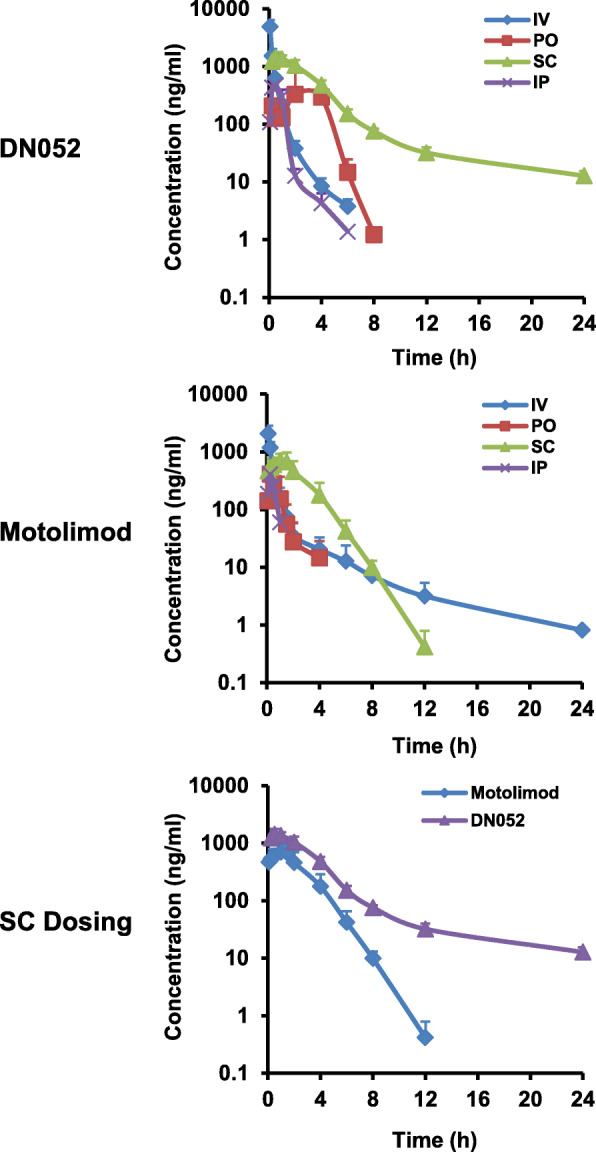
Comparison of PK with different dosing routes. DN052 and motolimod were iv, ip, sc or po administered to Sprague Dawley rats, respectively. Overall, sc appeared to have better PK than the other dosing routes for DN052. DN052 showed superior PK profile than motolimod

DN052 showed clean CYP profile with IC50 over 10 μM for all major CYP isoenzymes tested including 3A4, 1A2, 2C9, 2C19 and 2D6 (Fig. 1a). DN052 had favorable hERG parameter with IC50 over 30 μM whereas motolimod’s hERG IC50 was 3.84 μM (Fig. 1a) suggesting motolimod may have potential cardiac toxicity liability when used at high doses. Interestingly, the Cerep off-target screen also showed motolimod had effect on hERG (Supplementary Fig. 1 and 2). The observation of motolimod’s hERG effect in two independent assays strongly suggested motolimod’s cardiac liability may limit its dose levels. Comparison of PK parameters with different dosing routes including iv, ip, sc and po in rats revealed that sc dosing was more desirable (Table 1 and Fig. 2) and therefore sc dosing was selected in subsequent studies. Furthermore, DN052 showed superior in vivo PK profile than motolimod in rats and monkeys (Table 2). With sc administration, DN052 had higher AUC, Cmax, bioavailability and longer t_1/2_ than motolimod. The mouse PK of DN052 also showed favorable profiles (Table 2).

**Table 1:** Comparison of PK of DN052 with different dosing routes in rats

Animal ID	t_1/2_	T_max_	C_max_	AUC_(0-t)_	AUC_(0-∞)_	V_z_	CL_z_	MRT_(0-∞)_
	hr	hr	ng/L	ng/L*hr	ng/L*hr	L/kg	L/hr/kg	hr
IV (2.5 mg/kg)								
101	1.26	0.083	3790.58	1444.12	1446.82	3.15	1.73	0.46
102	1.28	0.083	6626.67	2156.96	2161.94	2.14	1.16	0.27
103	1.07	0.083	4242.69	1637.17	1642.71	2.36	1.52	0.42
Mean	1.21	0.083	4886.65	1746.08	1750.49	2.55	1.47	0.38
SD	0.12	0.000	1523.77	368.69	369.54	0.53	0.29	0.10
Animal ID	t_1/2_	T_max_	C_max_	AUC_(0-t)_	AUC_(0-∞)_	MRT_(0-∞)_	F	
	hr	hr	ng/L	ng/L*hr	ng/L*hr	hr	%	
SC (10 mg/kg)								
201	5.13	0.500	1832.27	5400.93	5472.83	3.31	77.33	
202	5.86	1.000	1544.70	4707.30	4823.60	3.95	67.40	
203	6.79	1.000	1383.32	5653.51	5799.55	4.23	80.95	
Mean	5.93	0.833	1586.76	5253.92	5365.33	3.83	75.22	
SD	0.83	0.289	227.41	489.94	496.78	0.48	7.01	
Animal ID	t_1/2_	T_max_	C_max_	AUC_(0-t)_	AUC_(0-∞)_	MRT_(0-∞)_	F	
	hr	hr	ng/L	ng/L*hr	ng/L*hr	hr	%	
PO (10 mg/kg)								
301	0.56	2.000	928.73	2104.83	2105.91	2.27	30.14	
302	0.48	4.000	374.47	916.24	916.99	3.73	13.12	
303	NA	4.000	309.82	917.56	NA	NA	13.14	
Mean	0.52	3.333	537.67	1312.88	1511.45	3.00	18.80	
SD	0.06	1.155	340.20	685.85	840.69	1.03	9.82

**Table 2:** PK profiles of DN052 and motolimod

Compound	Species	T_max_ h	Cmax (μg/mL)	AUC(0-t) (μg/mL*h)	AUC(0-∞) (μg/mL*h)	MRT_(0-∞)_h	t_1/2_ (h)	CL (iv) L/h/kg	Vz (iv) L/kg	F (%)
DN052	Mouse	0.50	4.26	8.62	8.83	3.65	6.30	0.87	0.81	77.18
	Rat	0.83	1.59	5.25	5.37	3.83	5.93	1.47	2.55	75.22
	Monkey	0.25	0.19	0.36	0.37	3.53	6.32	3.01	11.38	108.89
Motolimod	Rat	1.17	0.72	2.13	2.13	ND	0.92	2.73	18.64	57.40
	Monkey	2.00	0.08	0.27	0.27	2.95	1.43	ND	ND	ND
	Human^a^	0.53	1.52 ng/ml	2.99 ng/mL*h	ND	ND	1.73	ND	ND	ND

Mouse PK: iv dose at 2.5 mg/kg and sc dose at 10 mg/kg for DN052. Rat PK: iv dose at 2.5 mg/kg and sc dose at 10 mg/kg for both motolimod and DN052. Monkey PK: iv dose at 1 mg/kg for DN052; sc dose at 1 mg/kg for both motolimod and DN052. Human PK: sc dose at 0.1 mg/m^2^ for motolimod. ND: not determined. ^a^Data from published literature Northfelt, et al., [39]

### DN052 strongly inhibited tumor growth as a single agent or in combination with other anti-cancer drugs

Murine TLR8 was once thought to be non-functional. However, more recent studies including TLR8 knock-out mouse model studies indicated that murine TLR8 is functional and plays important roles in immune response albeit its receptor activity is lower in mice than in other species [41–43]. This limitation has been a major hurdle in drug discovery effort targeting TLR8. We addressed this limitation by using two different approaches: 1) Immune-competent mouse syngeneic tumor models: high doses of DN052 were used to offset the low TLR8 activity in rodents; 2) Human AML mouse xenograft model: human HL-60 AML cells could be directly targeted by TLR8 agonist through its effect of inducing terminal differentiation-mediated tumor suppression [24].

**Fig. 3:**
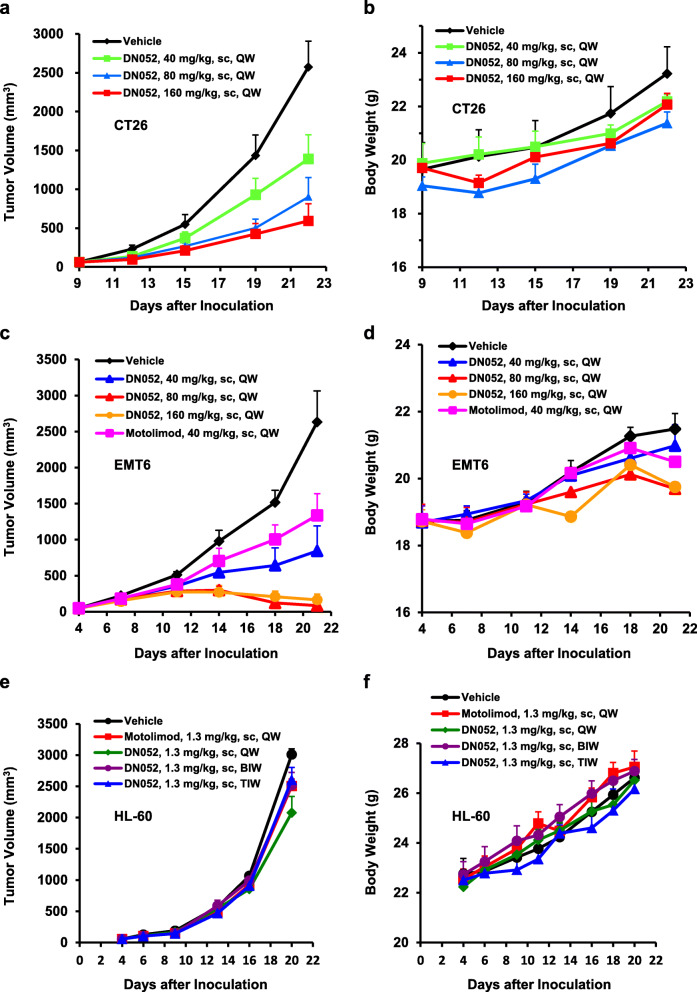
DN052 was more efficacious than motolimod and well-tolerated as a single agent in various mouse tumor models. DN052 treatment resulted in significant tumor growth inhibition in CT26 colon cancer (**a**, **b**), EMT6 breast cancer (**c**, **d**) and HL-60 AML (**e**, **f**) models. DN052 more strongly suppressed tumor growth than motolimod under the same conditions

DN052 strongly suppressed tumor growth in a dose dependent manner as a single agent when used at high doses at 40, 80 and 160 mg/kg in immune-competent CT26 mouse syngeneic colon cancer model (Fig. 3a). DN052 was well-tolerated in the mice at all the doses tested (Fig. 3b). Similar result was observed in the immune-competent EMT6 mouse syngeneic breast cancer model in which DN052 markedly suppressed tumor growth as a single agent and resulted in complete tumor regression in 1/8, 2/8 and 3/8 tumor-bearing mice at 40, 80 and 160 mg/kg, respectively (Fig. 3c, d). Moreover, when evaluated side-by-side under the same condition, DN052 appeared to be more efficacious than motolimod in EMT6 model (Fig. 3c). The strong efficacy produced by DN052 single agent suggested that DN052 had potential to be used as monotherapy in treating cancer patients.

**Fig. 4:**
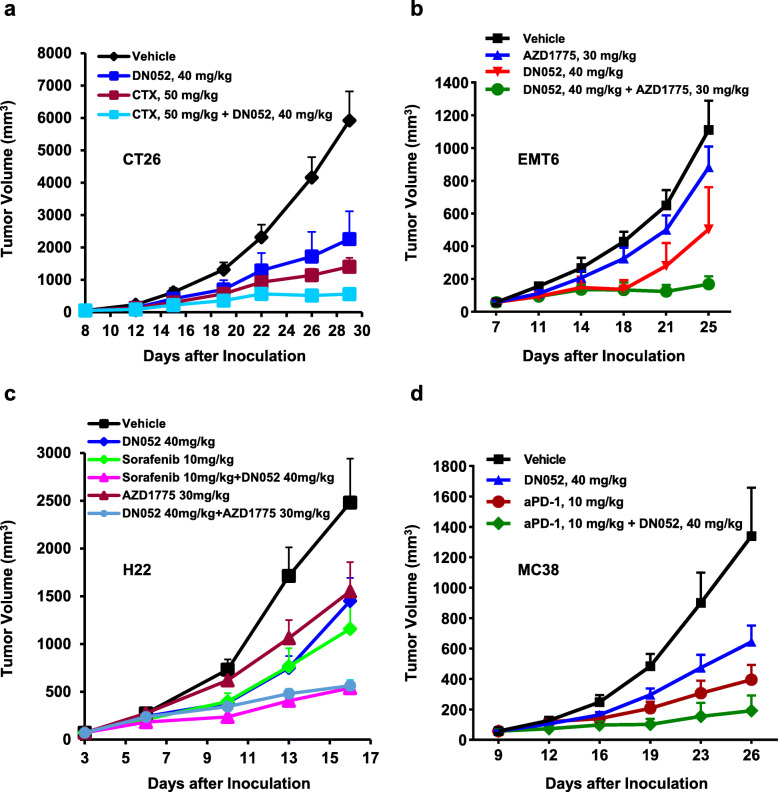
DN052 further reduced tumor growth when combined with other anti-cancer agents in mouse tumor models. Combination of DN052 and cyclophosphamide, WEE1 inhibitor AZD-1775, sorafenib or αPD-1 increased tumor suppression in several mouse tumor models: CT26 colon cancer model (**a**), EMT6 breast cancer model (**b**), H22 liver cancer model (**c**) and MC38 colon cancer model (**d**)

Furthermore, to explore its application in combination therapies addressing critical unmet medical needs, we carried out a series of combination studies in several cancer models. Combination of DN052 and the chemotherapeutic drug cyclophosphamide (CTX) resulted in stronger tumor suppression than either agent alone in immune-competent CT26 mouse syngeneic colon cancer model (Fig. 4a). In another combination study, while EMT6 model was largely resistant to AZD-1775, a WEE1 inhibitor currently in phase 2 clinical development [44], addition of DN052 drastically enhanced tumor growth inhibition compared to either agent alone (Fig. 4b). Similarly, combination of DN052 and AZD-1775 increased efficacy in immune-competent H22 mouse syngeneic liver cancer model (Fig. 4c). Moreover, addition of DN052 increased tumor growth inhibition produced by sorafenib, a standard of care (SoC) agent for liver cancer in H22 model (Fig. 4c). DN052 or anti-PD-1 monoclonal antibody (αPD-1) suppressed tumor growth as single agent in immune-competent MC-38 mouse syngeneic colon cancer model (Fig. 4d). Combination of DN052 and αPD-1 exhibited stronger tumor suppression than either agent alone though the effect of combination appeared less pronounced based on tumor growth inhibition rates (Fig. 4d). However, it is noteworthy that combination of DN052 and αPD-1 resulted in complete tumor regression in 1/7 MC-38 tumor-bearing mice whereas no complete tumor regression in mice treated with single agents further suggesting DN052 enhanced αPD-1’s anti-cancer effect (Fig. 4d).

Although the in vivo efficacy observed in the mouse syngeneic models was remarkable, the high doses used in these studies are less predictive of the human dose in clinical trials because of TLR8’s species difference in that murine TLR8’s activity is weaker than human TLR8 [41–43]. To gain further information about the in vivo efficacious doses more relevant to humans, we conducted a study using human HL-60 AML mouse xenograft model. DN052 impeded tumor growth in the immune-deficient mice bearing human HL-60 AML when dosed sc at 1.3 mg/kg. DN052 caused stronger tumor growth inhibition than motolimod under the same condition with tumor growth inhibition (TGI) rate 31% vs 17% indicating DN052 was more active in vivo than motolimod (Fig. 3e). The stronger activity of DN052 than motolimod was consistent with the results in the immune competent EMT6 mouse syngeneic model as well as the in vitro cell based assays. Furthermore, 1.3 mg/kg QW produced stronger efficacy than BIW and TIW indicating that infrequent dosing can be achieved. Importantly, DN052 appeared to be efficacious with all the dosing schedules tested. During the study, there was no mortality or significant changes in mouse body weight between test compounds-treated animals and the vehicle controls indicating all the treatment was well-tolerated by the animals (Fig. 3f). Based on these results and the published literature reporting QW dosing for motolimod [36], QW dosing schedule was chosen for DN052 in both efficacy and toxicology studies. Of note, since the HL-60 xenograft model used in this study was immune-deficient and very low dose of compounds was used, the anti-tumor activity of DN052 and motolimod observed was unlikely mediated through immune response. The in vivo result was further supported by the in vitro study in which HL-60 cell differentiation was more strongly induced by DN052 than motolimod (Supplementary Fig. 3). The anti-tumor effect was at least in part due to the direct effect on the human HL-60 cells through induction of leukemia cell terminal differentiation, which is also supported by the literature [24]. The relatively modest tumor growth inhibition observed in this model could be underestimated given the immune-deficient background where the TLR8’s immune modulation was largely bypassed in this model.

### DN052 induced pro-inflammatory cytokines

To understand the underlying mechanism for DN052’s anticancer effect, an ex vivo human PBMC assay was used to evaluate the immune modulatory activity of DN052. Motolimod was included as a reference compound. As shown in Table 3 and Supplementary Fig. 4, DN052 strongly induced the pro-inflammatory cytokines including TNF-α, IFN-α_2_, IL-1α, IL-1β, IL-6, IL-8, IL-10, IL-12p40, IL-12p70, MIP-1α, MIP-1β, G-CSF and IFN-γ. Overall, DN052 was more potent in inducing the cytokines than motolimod. Several of the cytokines showed robust induction for both DN052 and motolimod including MIP-1β, MIP-1α, IL-1β, IL-6, IL-8, IL-12p40 and TNF-α. Comparison of the EC_50_ values for these cytokines between DN052 and motolimod indicated that DN052 was about 7–22 times more potent than motolimod.

**Table 3:** DN052 induced cytokines in ex vivo human PMBCs

PD Marker	Donor	LPS	Motolimod	DN052
		EC50 (ng/ml)	EC50 (μM)	EC50 (μM)
MIP-1β	Donor 1	0.013	0.277	0.023
	Donor 2	0.012	0.248	0.028
MIP-1α	Donor 1	0.017	0.277	0.012
	Donor 2	0.007	0.208	0.013
IL-1α	Donor 1	0.799	0.849	0.056
	Donor 2	0.062	0.348	0.054
IL-12(p70)	Donor 1	0.483	0.285	ND^a^
	Donor 2	0.003	110.592	ND^a^
IFN-γ	Donor 1	108.800	4.157	0.050
	Donor 2	0.003	1.657	0.047
IL-10	Donor 1	1.366	0.304	0.012
	Donor 2	0.116	0.191	0.011
IL-1β	Donor 1	2.073	0.665	0.053
	Donor 2	0.077	0.556	0.051
IL-6	Donor 1	0.018	0.424	0.030
	Donor 2	0.013	0.439	0.034
IL-8	Donor 1	0.007	0.504	0.023
	Donor 2	0.006	0.502	0.023
TNF-α	Donor 1	0.036	0.321	0.048
	Donor 2	0.019	0.317	0.049
G-CSF	Donor 1	1.869	0.841	0.049
	Donor 2	0.042	0.461	0.049
IFN-α2	Donor 1	6.737	1.331	1.489
	Donor 2	ND^a^	0.344	0.505
IL-12(p40)	Donor 1	ND^a^	0.447	0.020
	Donor 2	ND^a^	0.344	0.047

^a^ND: not determined

To further investigate the immune response to DN052 in vivo, DN052 was sc administered to cynomolgus monkeys and changes in serum levels of cytokines IFN-γ, IL-1β, IL-6, IL-8, IL-10, IL-12p40, IL-12p70, TNF-α, G-CSF, MIP-1α, MIP-1β were measured. Consistent with the hPBMC result, treatment with DN052 resulted in strong induction of the cytokines. DN052 was more effective than motolimod when used at the same dose 1 mg/kg (Table 4).

**Table 4:** DN052 induced cytokines in monkeys

PD Marker	Timepoint (h)	Mean Values (pg/mL)			
		Motolimod (1 mg/kg, sc)	DN052 (1 mg/kg, sc)	DN052 (3 mg/kg, sc)	DN052 (10 mg/kg, sc)
IFN-γ	0	1.017	0.785	1.249	0.397
	6	0.500	39.126	9.894	9.544
	24	0.462	1.062	0.000	1.209
IL-10	0	0.271	0.295	0.366	0.217
	6	15.222	726.647	680.726	1497.839
	24	1.347	5.166	6.821	52.473
IL-1β	0	0.000	0.130	0.431	0.000
	6	0.286	2.196	3.085	4.071
	24	0.099	0.231	0.853	0.456
IL-6	0	1.510	3.403	7.566	2.574
	6	342.661	2747.061	1850.460	9266.511
	24	12.185	120.795	195.354	1464.089
IL-8	0	0.000	0.257	1.085	0.238
	6	0.378	1.215	1.964	5.416
	24	0.119	0.401	0.716	1.044
MIP-1α	0	71.636	56.670	58.578	65.268
	6	227.638	492.577	487.888	594.484
	24	87.417	152.799	151.320	181.215
MIP-1β	0	93.266	215.299	195.431	210.978
	6	1655.094	3190.677	1934.886	6215.896
	24	221.881	659.556	692.643	1158.540
IL-12p40	0	131.413	191.908	279.654	158.623
	6	305.212	7295.739	5927.984	5525.537
	24	118.409	653.428	553.852	633.985
G-CSF	0	7.135	29.193	41.588	29.765
	6	345.154	2411.265	2081.330	6683.544
	24	32.168	58.352	127.944	576.160
IL-12p70	0	2.582	2.458	2.214	ND^a^
	6	5.154	9.084	2.977	ND^a^
	24	3.305	2.834	0.000	ND^a^
TNF-α	0	ND^a^	ND^a^	1.931	3.033
	6	ND^a^	ND^a^	2.684	3.380
	24	ND^a^	ND^a^	1.676	2.501

^a^*ND* not determined

### DN052 had favorable safety profiles

The potential toxicity of DN052 when administered once weekly via subcutaneous injection to rodents (rats) or non-rodent large animals (monkeys) were investigated. In the Sprague Dawley rat GLP study, DN052 was dosed at 2, 6, 12 mg/kg, sc, QW, respectively. 12 mg/kg was determined to be no observed adverse effect level (NOAEL). Mild skin injection site reaction was observed. Maximum tolerated dose (MTD) was not reached. The strong tolerability exhibited by rats treated with DN052 was expected given TLR8’s species difference in that TLR8’s activity in rodents is very low [42]. In contrast, in the cynomolgus monkey GLP study, DN052 was dosed at 0.3, 0.6 and 2 mg/kg, sc, QW, respectively. Skin injection site reaction was observed at all dose levels. Microscopic examination at the terminal sacrifice showed minimal to slight acute or chronic-active inflammation, mixed cell infiltrates, and/or aggregation of foamy macrophages in the subcutis of animals administered 0.3 and 0.6 mg/kg and ulcer and/or acute inflammation in the lumbar skin/subcutis of animals administered 2.0 mg/kg. Microscopic changes at the injection sites of animals administered 0.3 or 0.6 mg/kg were considered non-adverse. At the recovery sacrifice, these microscopic findings had completely recovered indicating that the skin injection site reaction was reversible. 0.6 mg/kg was determined to be NOAEL and the highest non-severely toxic dose (HNSTD) is between 0.6 mg/kg and 2.0 mg/kg. No DN052-related changes in blood pressure were noted. No abnormal ECG waveforms or arrhythmias attributed to DN052 were observed in the cynomolgus monkeys administered 0.3, 0.6, or 2 mg/kg.

As reported in the literature, the highest dose of motolimod used in phase 1 clinical trials was 3.9 mg/m^2^ [39] and its phase 2 doses were 2.5–3.5 mg/m^2^ [33]. 3.9 mg/m^2^ in humans is equivalent to approximately 0.3 mg/kg in monkeys, which was half of the NOAEL of DN052 (0.6 mg/kg). Therefore, the toxicity result indicated that DN052 had favorable safety profile. On the other hand, the in vitro cell based assays showed that DN052 was 16-fold more potent than motolimod. The in vivo efficacy study in tumor models demonstrated that DN052 was more active than motolimod. Taken together, these results suggested that DN052 may have larger therapeutic index than motolimod in humans.

## Discussion

Harnessing the host’s immune system to eradicate cancer cells has become a powerful new approach to cancer therapy in recent years partly attributing to the unprecedented clinical success of immune checkpoint inhibitors [2, 3]. However, despite the significant progress in cancer immunotherapy with checkpoint blockade, majority of cancer patients do not respond to the current therapy [4]. The lack of response to immune checkpoint blockade in majority of cancer patients represents an urgent unmet medical need. Human body’s immune system consists of innate and adaptive immune pathways [1]. While releasing the immune checkpoint blockage in the adaptive immunity has proved effective therapy, targets involved in the innate immunity are just beginning to show promise in the fight against cancer [4]. We hypothesized that targeting innate immunity would enhance the anticancer efficacy produced by drugs targeting the adaptive immunity because of their complementary nature as host’s immune defense system.

TLR8 was chosen because it is one of the most important molecules of the innate immunity [42, 45–47]. Accumulating evidence indicated that activation of TLR8 could reverse Treg and MDSC mediated immune suppression resulting in strong tumor inhibition [15, 18–20]. One of the major causes of cancer immunotherapy failure is potent suppression of immune response by Treg or MDSC cells [20–22]. Therefore, TLR8 agonists possess the potential to turn immune unresponsive “cold” tumors to immune responsive “hot” tumors, thereby addressing the urgent unmet medical need in tumor immunotherapy.

DN052 is a novel TLR8 agonist displaying differentiated profiles compared to motolimod in that DN052 is more selective for TLR8 while sparing TLR7. Several TLR agonists including TLR4, 7, TLR7/8 dual and TLR9 agonists can be used mainly as a topical drug or dosed locally such as intratumoral injection because they are too toxic if used systemically [5]. This has been a major hurdle which limits their application in the clinic [12, 31, 32]. In contrast, our study demonstrated that the high selectivity of DN052 on TLR8 showed better safety allowing systemic dosing which was well tolerated in the in vivo studies. DN052 also showed stronger activity than motolimod, superior DMPK profiles in rats and monkeys and excellent PK in mice. TLR8 was previously thought to be non-functional in mice [42]. However, more recent studies indicated that TLR8 plays crucial roles in the immune response in mice albeit its receptor activity is much diminished in rodents including mice and rats compared to other species such as humans [41–43]. The species difference of TLR8 hindered much of the in vivo studies in the context of drug discovery. To address this challenge, we applied high doses of DN052 in syngeneic mouse tumor models to offset the reduced receptor activity of TLR8 in mice. DN052 showed strong in vivo efficacy when used as a single agent in immune-competent mouse syngeneic tumor models for multiple cancer types.

To understand the mechanism for its tumor suppression, ex vivo human PBMC and in vivo monkey studies were carried out and DN052 activated immune response evidenced by strong induction of the proinflammatory cytokines including TNF-α, IFN-α_2_, IL-1α, IL-1β, IL-6, IL-8, IL-10, IL-12p40, IL-12p70, MIP-1α, MIP-1β, G-CSF and INF-γ. Overall, DN052 was more potent than motolimod in inducing the cytokines including INF-γ, IL-12p40, TNF-α which were reported to be predictive of clinical benefit in motolimod-treated cancer patients [35] suggesting DN052 may have superior efficacy in humans.

In another study, immune-deficient mouse xenograft model carrying human HL-60 AML was used to test if DN052 could inhibit tumor growth in vivo at lower doses. In this model, DN052 exerted the anticancer activity mainly by directly targeting the human AML cells through induction of terminal differentiation-mediated tumor suppression [24]. The result showed that treatment with low dose of DN052 resulted in significant tumor inhibition in the human AML mouse xenograft model. This approach overcame the species limitation seen in the syngeneic mouse tumor models where very high doses of DN052 had to be used because lower doses didn’t produce anticancer efficacy due to the much diminished TLR8 receptor activity in mice. Furthermore, the result suggested that unlike the high doses of DN052 used in syngeneic mouse models, low doses of DN052 are expected to be efficacious in humans.

Collectively, the series of in vivo efficacy studies using various mouse models representing different cancer types suggested that DN052 may be useful for both solid tumors and hematological malignancies in the clinic.

After demonstrating DN052 was efficacious when used as a single agent, we went on to ask if combination of DN052 with another anticancer agent would enhance the efficacy of single agents. Interestingly, several agents including cyclophosphamide, sorafenib, WEE1 inhibitor AZD1775 and αPD-1 monoclonal antibody showed increased tumor growth inhibition when combined with DN052. Although the precise mechanism underlying the enhanced efficacy is not fully understood and awaits further investigation, some of the mechanistic insight can be deduced from the literature. For example, the increased tumor growth inhibition produced by combining DN052 and the chemotherapeutic cyclophosphamide was likely due to the increased tumor mutation load caused by alkylating DNA. In addition, earlier reports suggested that cyclophosphamide treatment could result in mobilization of immune cells which might also play a role in increasing the anticancer efficacy [48]. Combination of DN052 and WEE1 inhibitor showed potential synergy in suppressing tumor growth. The markedly enhanced efficacy was likely due to the enhanced antigen presentation as a result of higher tumor mutation load considering the function of WEE1 in DNA damage and repair [49]. The combination therapy data of DN052 and αPD-1 supported the hypothesis that targeting both innate and adaptive immunity could increase anticancer efficacy. Taken together, the in vivo efficacy results strongly suggested that DN052 has the potential to be used as a backbone either as a single agent or in combination therapy in the clinic.

In clinical trials, motolimod is also administered subcutaneously and it has been reported that the skin injection site reaction (ISR) was correlated with significantly longer survival in terms of progression free survival (PFS) and overall survival (OS) in both ovarian, and head and neck cancer patients [35, 36]. In preclinical studies, DN052 caused ISR more consistently than motolimod under the same conditions in animal studies suggesting DN052 may lead to better clinical outcome in human patients and ISR could be used as a surrogate marker in DN052 clinical trials. In addition, human papillomavirus (HPV)-positive status was shown to correlate with longer survival in head and neck cancer patients treated with motolimod [36]. The high prevalence of HPV-positive head and neck cancer patients represents significant clinical indication. Therefore, HPV status may be useful in DN052 clinical trials.

DN052 is currently advancing in phase 1 trials in cancer patients in the US (ClinicalTrials.gov Identifier: NCT03934359) and both the safety and efficacy will be rigorously tested in the clinic. It is noteworthy that besides cancer indications, TLR8 agonists such as DN052 can be used for other indications including cancer vaccines [50] and the treatment of viral infections including HBV.

## Materials and methods

### In vitro experiments

HEK-Blue™ hTLR4, 7, 8 and 9 cell lines were purchased from InvivoGen (Hong Kong). The cells express the human TLR gene and NF-κB/AP-1-inducible SEAP (secreted embryonic alkaline phosphatase) reporter gene. SEAP levels produced upon TLR stimulation can be determined by QUANTI-Blue™. In the cell-based assay, the main reagents used were QUANTI-Blue™ (InvivoGen) and ATPlite 1 Step (Perkin Elmer). The main instruments used were the microplate reader SpectraMax 340PC (Molecular Device) and Envision (Perkin Elmer). Motolimod and DN052 were synthesized in house. The chemical structure and synthesis of DN052 and related compounds were described in our published patent 10,669,252. The compounds were in 10 mM stock solution in DMSO and stored at − 20°C. The compounds were serially diluted in the concentration range from 0.5 nM to 15 μM (up to 50 μM in the hTLR4, hTLR7 and hTLR9 assays) and added to 96-well plates. DMSO was used as the negative control. LPS-EK, R848, motolimod and ODN2006 were used as the positive control for TLR4, 7, 8 and 9, respectively. The cells were cultured and treated with the compounds at 37°C, 5%CO_2_ for 24 h. After 24 h of incubation, 20 μl of the supernatant of each well was added to 180 μl of QUANTI-Blue™. The plates were incubated at 37°C for 1.5 h. After 1.5 h of incubation, the optical density was measured using the spectrophotometer at 650 nm in the microplate reader of SpectraMax 340PC. The cell viability of each well was determined using ATPlite 1 Step following the manufacturer’s instruction. The luminescence signal in each well of plates was measured in the microplate reader of Envision.

To assess the potential off-target effects of DN052 relative to motolimod, 10 μM of compounds were tested in Eurofins Cerep44 panel following the manufacturer’s instruction. Compound binding was calculated as a % inhibition of the binding of a radioactively labeled ligand specific for each target. Compound enzyme inhibition effect was calculated as a % inhibition of control enzyme activity.

### DMPK and hERG assays

CYP inhibition assay was conducted to evaluate the inhibitive potential of the compounds on CYP 1A2, 2C9, 2C19, 2D6 and 3A4 using human liver microsomes (BD Gentest). Curve-fitting was performed to calculate IC50 using a Sigmoidal (non-linear) dose-response model using GraphPad Prism (GraphPad Software Inc.).

To determine the in vivo pharmacokinetic profiles, the compounds were formulated with 11% captisol and then administered iv, ip, sc and po into Sprague Dawley rats or iv and sc into mice or cynomolgus monkeys, respectively. Blood samples were collected before dosing and 0.083 h, 0.25 h, 0.5 h, 1 h, 2 h, 4 h, 6 h, 8 h, 12 h and 24 h after dosing. Blood samples were collected in tubes containing K_2_EDTA. Plasma was isolated from the blood samples by centrifugation of the blood samples at 2400 × g for 5 min at 4°C. Plasma samples were measured using LC-MS/MS (Waters ACQUITY UPLC and TQ 6500+) to determine the concentration of compounds. The data was analyzed using WinNonlin 7.0 to calculate the PK parameters such as AUC_0-t_, AUC_0-∞_, MRT_0-∞_, Cmax, Tmax, t_1/2_ and F.

hERG assay was conducted using CHO cell line stably transduced with hERG cDNA. Whole-cell recordings were performed using automated QPatch (Sophion Biosciences). Data were analyzed using Assay Software provided by Sophion (assay software V5.0), Microsoft Excel and GraphPad Prism.

### In vivo efficacy studies

All the animal experiments were conducted following Association for Assessment and Accreditation of Laboratory Animal Care (AAALAC)‘s guideline. The animal protocols were approved by the Institutional Animal Care and Use Committee (IACUC). Cell lines were purchased from credible vendors including ATCC. After tumor cell inoculation, the animals were checked daily for morbidity and mortality. At the time of routine monitoring, the animals were checked for any effects of tumor growth and treatment on the animals’ well-being such as mobility, food and water consumption, body weight gain/loss, matting and any other abnormalities. Clinical observations were recorded. Tumor volumes were measured twice a week in two dimensions using a caliper and the volume was expressed in mm^3^ using the formula: V = 0.5 a × b^2^ where a and b are the long and short diameters of the tumor, respectively. The procedures of dosing as well as tumor and body weight measurement were conducted in a laminar flow cabinet.

For CT26 experiments, CT26 mouse colon cancer cells were cultured in vitro as a monolayer culture in RPMI1640 medium supplemented with 10% fetal bovine serum and 1% penicillin/streptomycin at 37°C in an atmosphere of 5% CO_2_ in air. The CT26 cells were routinely passaged twice per week by trypsin-EDTA treatment. The cells growing in the exponential growth phase were harvested and counted for tumor inoculation. 6–8 weeks old female Balb/c mice were purchased from Shanghai Lingchang Bio-Technology Co. Ltd. (Shanghai, China). Each mouse was inoculated subcutaneously at the right flank region with 1 × 10^5^ CT26 tumor cells in 100 μl ice-cold PBS. The tumor-bearing mice were randomized into different groups with 6 mice per group. Treatment started when the mean tumor volume reached 62 mm^3^. DN052 was formulated in 11% captisol and then subcutaneously (sc) administered once a week to the tumor-bearing mice at 40, 80 and 160 mg/kg, respectively. In the combination study in CT26 model, the mice were randomized into groups with 7 mice per group. Treatment started when the mean tumor volume reached 54 mm^3^. DN052 was sc administered at 40 mg/kg once a week. 50 mg/kg of cyclophosphamide (CTX) was intraperitoneally (ip) dosed once a week to CT26 tumor-bearing mice. Vehicle treated mice were included as controls.

For EMT6 experiments, EMT6 mouse breast cancer cells were cultured in vitro as a monolayer culture in RPMI1640 medium supplemented with 10% fetal bovine serum and 1% penicillin/streptomycin at 37°C in an atmosphere of 5% CO_2_ in air. The tumor cells were routinely passaged twice per week by trypsin-EDTA treatment. The cells growing in the exponential growth phase were harvested and counted for tumor inoculation. 6–8 weeks old female Balb/c mice were purchased from Beijing Vital River Laboratory Animal Technology Co., Ltd. (Beijing, China). Each mouse was inoculated subcutaneously at the right flank region with 1 × 10^5^ EMT6 tumor cells in 100 μl ice-cold PBS. The tumor-bearing mice were randomized into different groups with 8 mice per group. Treatment started when the mean tumor volume reached 58 mm^3^. DN052 was sc administered once a week to the tumor-bearing mice at 40, 80 and 160 mg/kg, respectively. Motolimod was sc administered once a week at 40 mg/kg. In the combination study in EMT6 model, the mice were randomized into groups with 6 mice per group. Treatment started when the mean tumor volume reached 58 mm^3^. DN052 was sc administered at 40 mg/kg once a week. AZD-1775 was dosed at 30 mg/kg, oral (po), bid to EMT6 tumor-bearing mice. Vehicle treated mice were included as controls.

For H22 experiment, H22 mouse liver cancer cells were cultured in vitro as a monolayer culture in RPMI1640 medium supplemented with 10% fetal bovine serum and 1% penicillin/streptomycin at 37°C in an atmosphere of 5% CO_2_ in air. The tumor cells were routinely passaged twice per week by trypsin-EDTA treatment. The cells growing in the exponential growth phase were harvested and counted for tumor inoculation. 6–8 weeks old female Balb/c mice were purchased from Beijing Vital River Laboratory Animal Technology Co., Ltd. (Beijing, China). Each mouse was inoculated subcutaneously at the right flank region with 6 × 10^5^ H22 tumor cells in 100 μl ice-cold PBS. The tumor-bearing mice were randomized into different groups with 8 mice per group. Treatment started when the mean tumor volume reached 72 mm^3^. DN052 was sc administered at 40 mg/kg once a week. Sorafenib was administered at 10 mg/kg, po, bid. AZD-1775 was dosed at 30 mg/kg, po, bid. Vehicle treated mice were included as controls.

For MC38 experiment, MC38 mouse colon cancer cells were cultured in vitro as a monolayer culture in DMEM medium supplemented with 10% fetal bovine serum and 1% penicillin/streptomycin at 37°C in an atmosphere of 5% CO_2_ in air. The tumor cells were routinely passaged twice per week by trypsin-EDTA treatment. The cells growing in the exponential growth phase were harvested and counted for tumor inoculation. 7–8 weeks old female C57BL/6 mice were purchased from Beijing Vital River Laboratory Animal Technology Co., Ltd. (Beijing, China). Each mouse was inoculated subcutaneously at the right flank region with 5 × 10^5^ MC38 tumor cells in 100 μl ice-cold PBS. The tumor-bearing mice were randomized into different groups with 7 mice per group. Treatment started when the mean tumor volume reached 57 mm^3^. DN052 was sc administered at 40 mg/kg once a week. The anti-PD-1 monoclonal antibody αPD-1 (Clone RMP1–14, Bioxcell) was administered at 10 mg/kg, ip, once a week. Vehicle treated mice were included as controls.

For HL-60 experiment, HL-60 human acute promyelocytic leukemia (AML) cells were cultured in vitro in suspension in RPMI1640 medium supplemented with 10% fetal bovine serum and 1% penicillin/streptomycin at 37°C in an atmosphere of 5% CO_2_ in air. The tumor cells were routinely passaged twice per week. The cells growing in the exponential growth phase were harvested and counted for tumor inoculation. 6–7 weeks old female NOD/SCID mice were purchased from HFK Bio-Technology Co. Ltd. (Beijing, China). Each mouse was inoculated subcutaneously at the right flank region with 1 × 10^7^ HL-60 cells in 100 μl ice-cold PBS. The tumor-bearing mice were randomized into different groups with 6 mice per group. Treatment started when the mean tumor volume reached 56 mm^3^. 1.3 mg/kg of DN052 was sc administered once a week (qw), twice a week (biw) or three times a week (tiw), respectively. 1.3 mg/kg of motolimod was sc administered once a week. Vehicle treated mice were included as controls.

### Cytokine induction assays

To evaluate the activity of DN052 and motolimod in immune modulation, cytokine induction experiment was conducted in human peripheral blood mononuclear cells (PBMCs). Briefly, PBMCs were isolated from fresh human blood (Chempartner, Shanghai, China). 2 × 10^5^/100 μL/well hPBMCs were plated on 96-well cell culture plates. Serial dilutions of compounds with concentration range from 2 nM to 30 μM were added in duplicate wells. LPS was included as a control. The plate was incubated in a 37°C, 5% CO_2_ incubator for 24 h. The supernatant was harvested for cytokine detection using MILLIPLEX MAP Human Cytokine/Chemokine Magnetic Bead Panel - Immunology Multiplex Assay (Millipore) following the manufacturer’s protocol. Cytokines including TNF-α, IFN-α2, IL-1α, IL-1β, IL-6, IL-8, IL-10, IL-12p40, IL-12p70, MIP-1α, MIP-1β, G-CSF and IFN-γ were measured.

The cytokine induction activity of DN052 was further investigated in vivo using cynomolgus monkeys. Cynomolgus monkeys were purchased from Guangxi Xiongsen Primate Laboratory Animals Co., Ltd. (Guangxi, China) and three 3–4 years old male monkeys were used in each group. A single dose of DN052 was sc administered to monkeys at 1, 3 and 10 mg/kg, respectively. A single dose of motolimod was sc administered at 1 mg/kg. Blood samples were collected pre-dose, 6 and 24 h post-dose. Levels of cytokines including IFN-γ, IL-10, IL-1β, IL-6, IL-8, MIP-1α, MIP-1β, G-CSF, IL-12p40, IL-12p70 and TNF-α were measured using MSD ECL cytokine assay kit following the manufacturer’s protocol.

### Toxicology and safety pharmacology studies

Toxicity of DN052 was evaluated in rats and cynomolgus monkeys. First, Non-Good Laboratory Practice (Non-GLP) dose tolerability study was performed to find appropriate dose ranges. DN052 was sc administered to rats and monkeys and standard toxicology endpoints were evaluated. Then, GLP toxicology study was conducted. All aspects of the GLP studies were conducted in accordance with China Food and Drug Administration Good Laboratory Practice for Nonclinical Laboratory Studies and the United States Food and Drug Administration (FDA) Good Laboratory Practice (GLP) for Nonclinical Laboratory Studies. In the rat GLP study, DN052 was sc administered once a week for 3 weeks to Sprague Dawley rats at 2, 6 and 12 mg/kg, respectively, followed by a 4-week recovery phase. Vehicle treated rats were included as controls. Seven to nine weeks old rats were purchased from Vital River Laboratory Animal Technology Co. Ltd. (Beijing, China) and 30 rats (15 males and 15 females) were used in each group. Assessment of toxicity was based on mortality, clinical observations, skin injection site reaction, body weight, food consumption, ophthalmic observations and clinical and anatomic pathology. In the monkey GLP study, DN052 was sc administered once a week for 3 weeks to cynomolgus monkeys at 0.3, 0.6 and 2 mg/kg, respectively, followed by a 4-week recovery phase. Vehicle treated monkeys were included as controls. Three to four years old cynomolgus monkeys were purchased from Suzhou Xishan ZhongKe Laboratory Animal Co., Ltd. (Suzhou, Jiangsu, China) and 10 monkeys (5 males and 5 females) were used in each group. Assessment of toxicity was based on mortality, clinical observations, skin injection site reaction, body weight, food consumption, ophthalmology, vital signs (including body temperature, blood pressure and respiration rate), electrocardiography (ECG) and clinical and anatomic pathology. In the GLP safety pharmacology study, animals were monitored for cardiovascular function in cynomolgus monkeys administered with 0.3, 0.6 and 2 mg/kg of DN052.

### Statistical analyses

One-way ANOVA followed by Dunnett’s post-test and paired t-test were applied to assess the statistical significance of differences between treatment groups using GraphPad Prism. Statistical significance was accepted when *p* < 0.05.

## Supplementary information

**Additional file 1: Supplementary Fig. 1.** Cerep screen showed less off-target effects of DN052 compared to motolimod. **Suplementary Fig.** **2****.** Full panels of Cerep screen revealed cleaner off-target profile of DN052 than motolimod. Values higher than 50% were considered to represent significant effects of the test compounds. a, Binding assays were performed on 38 targets. b, Enzyme and uptake assays were performed on 6 targets. **Supplementary Fig. 3.** Nitroblue Tetrazolium (NBT) cell differentiation assay. The cell differentiation assay was performed using HL-60 cells as described in Ignatz-Hoover et al. [24] and the results showed DN052 was more active than motolimod in inducing HL-60 cell differentiation. **Supplementary Fig. 4.** Representative data from human PBMC assay. MIP-1β was induced by LPS, motolimod and DN052, respectively, in the ex vivo human PMBC assay. DN052 more strongly induced the cytokine than motolimod. Similar results were obtained from two different donors. The concentrations of LPS were in ng/ml whereas the concentrations of motolimod and DN052 were in μM.

## Data Availability

Data sharing not applicable to this article as no datasets were generated or analyzed during the current study.

## Abbreviations

ADMET: absorption, distribution, metabolism, excretion and toxicity
CC50: 50% cytotoxicity concentration
DMPK: drug metabolism and pharmacokinetics
DMSO: dimethyl sulfoxide
EC50: 50% effective concentration
GLP: good laboratory practice
IC50: 50% inhibitory concentration
IP: intraperitoneally
IV: intravenously
MDSC: myeloid-derived suppressor cell
OD: optical density
PAMPs: pathogen associated molecular patterns
PBMC: peripheral blood mononuclear cell
PO: orally (from Latin “per os”, by mouth)
SC: subcutaneously
TLRs: toll-like receptors
