# Correction to: Development of a novel TLR8 agonist for cancer immunotherapy

**DOI:** 10.1186/s43556-021-00042-3

**Published:** 2021-05-21

**Authors:** Yuxun Wang, Heping Yang, Huanping Li, Shuda Zhao, Yikun Zeng, Panpan Zhang, Xiaoqin Lin, Xiaoxiang Sun, Longsheng Wang, Guangliang Fu, Yaqiao Gao, Pei Wang, Daxin Gao

**Affiliations:** Shanghai Denovo Pharmatech Co., Ltd., 576 Libing Road, Shanghai Zhangjiang High-Tech Park, Pudong New District, Shanghai, 201203 China

**Correction to: Mol Biomed 1, 6 (2020)**

**https://doi.org/10.1186/s43556-020-00007-y**

In article [1], the authors would like to change the Competing Interests statement to below:

**Competing interests**

YW, HY and DG have patent 10669252 (Benzazepine derivative, preparation method, pharmaceuticalcomposition and use thereof). YW, HY, HL, SZ, YZ, PZ, XL, XS, LW, GF, YG, PW and DG are the employees of Shanghai Denovo Pharmatech Co., Ltd.. This work was supported by Shanghai Denovo Pharmatech Co., Ltd..
