# Supplementary material for: Development of a novel TLR8 agonist for cancer immunotherapy
Source: Mol Biomed. 2020 Sep 10;1:6. doi: 10.1186/s43556-020-00007-y (PMC8607422; doi:10.1186/s43556-020-00007-y)
Supplement: Supplementary file 1 — Additional file 1: Supplementary Fig. 1. Cerep screen showed less off-target effects of DN052 compared to motolimod. Suplementary Fig. 2. Full panels of Cerep screen revealed cleaner off-target profile of DN052 than motolimod. Values higher than 50% were considered to represent significant effects of the test compounds. a, Binding assays were performed on 38 targets. b, Enzyme and uptake assays were performed on 6 targets. Supplementary Fig. 3. Nitroblue Tetrazolium (NBT) cell differentiation assay. The cell differentiation assay was performed using HL-60 cells as described in Ignatz-Hoover et al. [24] and the results showed DN052 was more active than motolimod in inducing HL-60 cell differentiation. Supplementary Fig. 4. Representative data from human PBMC assay. MIP-1β was induced by LPS, motolimod and DN052, respectively, in the ex vivo human PMBC assay. DN052 more strongly induced the cytokine than motolimod. Similar results were obtained from two different donors. The concentrations of LPS were in ng/ml whereas the concentrations of motolimod and DN052 were in μM. [file 43556_2020_7_MOESM1_ESM.pdf]

Supplementary Data

Motolimod

Assay	1.0E-05 M
A_{2A}*(h)* (agonist radioligand)	52.1%
α_{1A}*(h)* (antagonist radioligand)	76.6%
α_{2A}*(h)* (antagonist radioligand)	84.9%
D_1*(h)* (antagonist radioligand)	77.6%
5-HT_{2A}*(h)* (agonist radioligand)	73.4%
5-HT_{2B}*(h)* (agonist radioligand)	98.3%
Ca^{2+} channel (L, dihydropyridine site) (antagonist radioligand)	85.8%
Potassium Channel hERG (human)- [3H] Dofetilide	59.2%
Na^+ channel (site 2) (antagonist radioligand)	89%
norepinephrine transporter*(h)* (antagonist radioligand)	67.7%
acetylcholinesterase *(h)*	83.5%

DN052

Assay	1.0E-05 M
α_{1A}*(h)* (antagonist radioligand)	75.3%
α_{2A}*(h)* (antagonist radioligand)	80.9%
D_1*(h)* (antagonist radioligand)	77.3%
H_1*(h)* (antagonist radioligand)	55.1% (motolimod at 29.1%)
M_2 *(h)* (antagonist radioligand)	56% (motolimod at 44%)
5-HT_{2A}*(h)* (agonist radioligand)	87.2%
5-HT_{2B}*(h)* (agonist radioligand)	101.8%
Na^+ channel (site 2) (antagonist radioligand)	68.1%
acetylcholinesterase *(h)*	97.3%

Supplementary Fig. 1 Cerep screen showed less off-target effects of DN052 compared to motolimod.

a

Targets Motolimod DN052

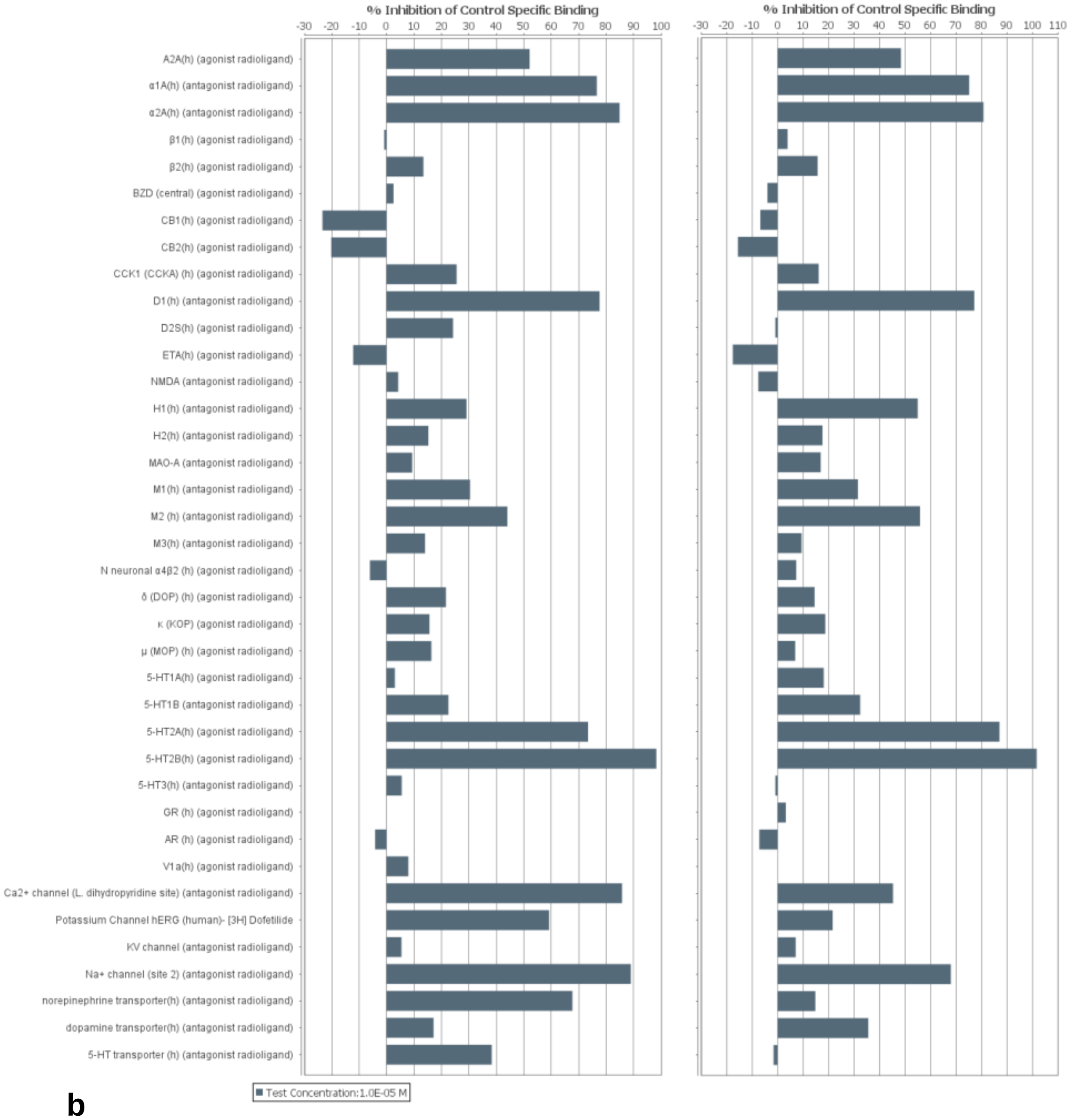

b

Targets Motolimod DN052

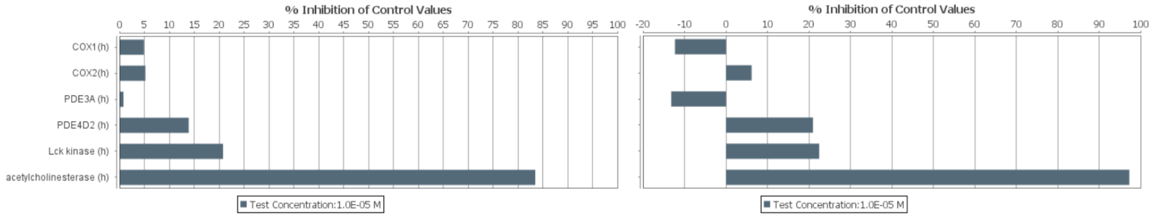

Supplementary Fig. 2 Full panels of Cerep screen revealed cleaner off-target profile of DN052 than motolimod. Values higher than 50% were considered to represent significant effects of the test compounds. a, Binding assays were performed on 38 targets. b, Enzyme and uptake assays were performed on 6 targets.

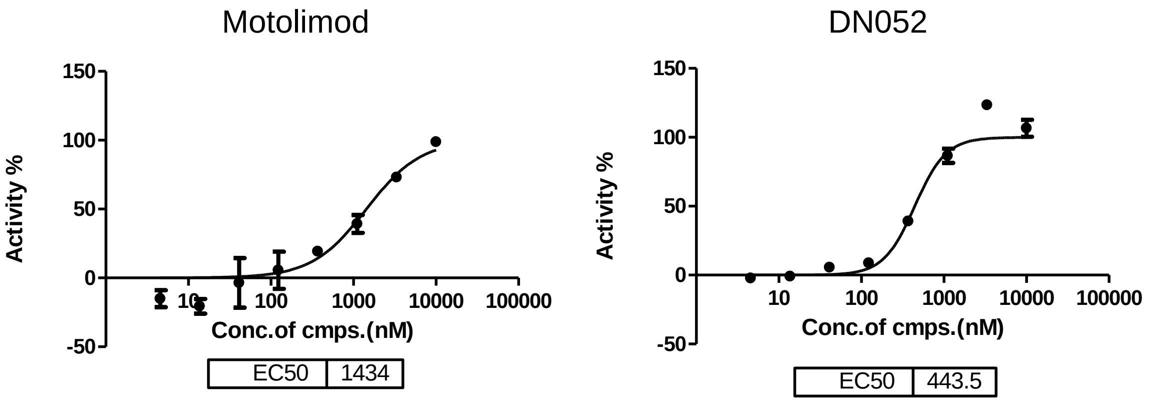

Supplementary Fig. 3 Nitroblue Tetrazolium **(**NBT) cell differentiation assay. The cell differentiation assay was performed using HL-60 cells as described in Ignatz-Hoover et al. 2015 and the results showed DN052 was more active than motolimod in inducing HL-60 cell differentiation.

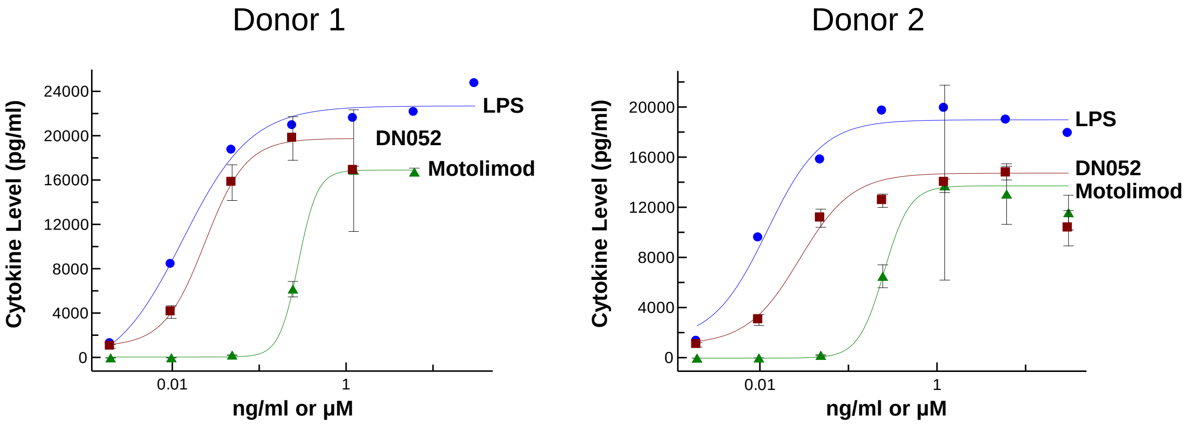

Supplementary Fig. 4 Representative data from human PBMC assay. MIP-1β was induced by LPS, motolimod and DN052, respectively, in the ex vivo human PMBC assay. DN052 more strongly induced the cytokine than motolimod. Similar results were obtained from two different donors. The concentrations of LPS were in ng/ml whereas the concentrations of motolimod and DN052 were in µM.
